# Endogenous reference RNAs for microRNA quantitation in formalin-fixed, paraffin-embedded lymph node tissue

**DOI:** 10.1038/s41598-018-24338-7

**Published:** 2018-04-12

**Authors:** Katsushige Inada, Yasushi Okoshi, Yukiko Cho-Isoda, Shingo Ishiguro, Hisashi Suzuki, Akinori Oki, Yoshio Tamaki, Toru Shimazui, Hitoaki Saito, Mitsuo Hori, Tatsuo Iijima, Hiroshi Kojima

**Affiliations:** 1 Department of Hematology, Ibaraki Prefectural Central Hospital, Ibaraki, Japan; 2 Ibaraki Clinical Education and Training Center, Faculty of Medicine, University of Tsukuba, Ibaraki, Japan; 3 Department of Medical Oncology, Ibaraki Prefectural Central Hospital, Ibaraki, Japan; 4 Department of Thoracic Surgery, Ibaraki Prefectural Central Hospital, Ibaraki, Japan; 5 Department of Obstetrics and Gynecology, Ibaraki Prefectural Central Hospital, Ibaraki, Japan; 6 Department of Radiation Oncology, Ibaraki Prefectural Central Hospital, Ibaraki, Japan; 7 Department of Urology, Ibaraki Prefectural Central Hospital, Ibaraki, Japan; 8 Department of Pathology, Ibaraki Prefectural Central Hospital, Ibaraki, Japan

## Abstract

Lymph node metastasis is one of the most important factors for tumor dissemination. Quantifying microRNA (miRNA) expression using real-time PCR in formalin-fixed, paraffin-embedded (FFPE) lymph node can provide valuable information regarding the biological research for cancer metastasis. However, a universal endogenous reference gene has not been identified in FFPE lymph node. This study aimed to identify suitable endogenous reference genes for miRNA expression analysis in FFPE lymph node. FFPE lymph nodes were obtained from 41 metastatic cancer and from 16 non-cancerous tissues. We selected 10 miRNAs as endogenous reference gene candidates using the global mean method. The stability of candidate genes was assessed by the following four statistical tools: BestKeeper, geNorm, NormFinder, and the comparative ΔCt method. miR-103a was the most stable gene among candidate genes. However, the use of a single miR-103a was not recommended because its stability value exceeded the reference value. Thus, we combined stable genes and investigated the stability and the effect of gene normalization. The combination of miR-24, miR-103a, and let-7a was identified as one of the most stable sets of endogenous reference genes for normalization in FFPE lymph node. This study may provide a basis for miRNA expression analysis in FFPE lymph node tissue.

## Introduction

Formalin fixation and paraffin embedding is the universal standard pathological technique for preserving tissue. These formalin-fixed, paraffin-embedded (FFPE) tissue samples are the most readily available, archival material for discovering biomarkers and validating human diseases; however, formalin fixation and paraffin embedding induces extensive degradation of nucleic acids such as DNA or mRNA^1,2^. MicroRNAs (miRNAs), a class of non-coding small RNAs (approximately 22 nucleotides), are relatively well preserved in FFPE tissue. Previous studies have revealed a positive correlation in miRNAs expression between FFPE tissues and frozen cells/tissues^3–5^. miRNAs regulate post-transcriptional gene expression by binding to the complementary sites of target mRNAs and have important functions in oncogenesis or as tumor suppressor due to their prominent role in cancer pathway regulation^6^. Because of their stability in FFPE tissue, miRNAs can serve as invaluable cancer biomarkers.

Quantitative real-time PCR (qRT-PCR) using the threshold cycle (Ct) value is one of the most sensitive methods for miRNA detection and quantification. In this method, an endogenous reference gene, which is stably expressed in every sample, regardless of the pathogenesis or diagnosis, is used for normalization. Careful validation of the endogenous reference gene is crucial for obtaining accurate data regarding miRNA expression^7^. To the best of our knowledge, a universal reference gene that is suitable for all FFPE tissues has not been found. For this reason, appropriate reference miRNAs should be selected for specific experiments, target cells, or tissues of interest. A stable endogenous reference gene should have high and constant level of expression in all FFPE tissue samples.

In this study, we investigated candidate miRNA reference genes in lymph nodes. Lymph node biopsy is generally performed to diagnose lymph node swelling, particularly when malignancy is suspected, and this procedure is also employed to determine the tissue type of metastatic carcinoma. To date, a miRNA expression profile can be used to identify the tissue of origin of carcinoma of unknown primary thanks to its tissue specificity^8,9^.

The present study aimed to find specific miRNAs that could serve as reliable and reproducible endogenous reference for FFPE lymph node tissue and to promote the further application of miRNA analysis. First, 10 miRNAs were selected as endogenous reference gene candidates from 71 small RNAs (Table 1). These consisted of 8 genes (miR-16, miR-24, miR-103a, miR-191, let-7a, U6 snRNA, SNORD44, and SNORD48), which are commonly used as reference in cancer studies^3,7,10–12^; 48 cancer-associated genes which have been used to identify cancer tissue origin^8^; and 15 genes of interest, which may be involved in cancer development. Then, the expression stability of the candidate genes was assessed using four statistical tools: BestKeeper^13^, geNorm^14^, NormFinder^15^, and the comparative ΔCt method^16^. Finally, stable gene sets were evaluated by comparing them with other normalization factors such as small nucleolar RNAs.

**Table 1:** The small RNAs used in this study.

Gene name	SD (ΔCt_Glo_)	Gene name	SD (ΔCt_Glo_)	Gene name	SD (ΔCt_Glo_)	No.
hsa-miR-191-5p^†^	0.67^*^	hsa-let-7a-5p^†^	0.84^*^	hsa-miR-193b-3p^†^	1.09	0
hsa-miR-24-3p^†^	0.68^*^	hsa-miR-34a-5p^†^	0.84^*^	hsa-miR-423-5p^†^	1.10	
hsa-miR-152-3p^†^	0.72^*^	hsa-miR-21-5p^†^	0.95^*^	hsa-miR-29c-3p^†^	1.12	
hsa-miR-148b-3p^†^	0.73^*^	hsa-miR-16-5p^†^	0.98^*^	hsa-let-7e-5p^†^	1.16	
hsa-miR-103a-3p^†^	0.79^*^	hsa-miR-29b-3p^†^	1.05	SNORD44^†^	1.19	
hsa-miR-92a-3p^†^	0.82^*^	hsa-miR-92b-3p^†^	1.05			
hsa-let-7i-5p^†^	0.69	hsa-miR-193a-3p^†^	1.06	hsa-miR-145-5p^†^	1.81	1
hsa-miR-181b-5p^†^	0.70	hsa-miR-29a-3p^†^	1.07	hsa-miR-135a-5p^†^	1.83	
hsa-miR-106b-5p^†^	0.76	hsa-miR-214-3p^†^	1.22	hsa-miR-31-5p^†^	1.84	
hsa-miR-345-5p^†^	0.86	hsa-miR-130a-3p^†^	1.36	hsa-miR-192-5p^†^	1.91	
hsa-miR-27b-3p^†^	0.94	hsa-miR-363-3p^†^	1.36	hsa-miR-194-5p^†^	1.92	
hsa-miR-99a-5p^†^	0.98	hsa-miR-142-3p^†^	1.55	hsa-miR-210-3p^†^	1.95	
hsa-miR-132-3p^†^	1.03	hsa-miR-181a-5p^†^	1.60			
SNORD48^†^	1.06	hsa-miR-146a-5p^†^	1.65			
hsa-miR-19b-3p^†^	0.97	U6 snRNA^†^	1.84	hsa-miR-155-5p^†^	1.24	≥2
hsa-miR-10b-5p^†^	1.50	hsa-miR-138-5p^†^	2.08	hsa-miR-148a-3p^†^	1.30	
hsa-miR-196a-5p^†^	2.69	hsa-miR-141-3p^†^	3.52	hsa-miR-10a-5p^†^	1.35	
hsa-miR-200c-3p^†^	3.39	hsa-miR-27a-3p^†^	1.09	hsa-miR-182-5p^†^	2.25	
hsa-miR-153-3p	1.49	hsa-miR-592	2.22	hsa-miR-122-5p	2.60	≥6
hsa-miR-372-3p	1.57	hsa-miR-124-3p	2.30	hsa-miR-509-3p	2.68	
hsa-miR-204-5p	1.62	hsa-miR-187-3p	2.33	hsa-miR-200a-3p	2.96	
hsa-miR-572	1.84	hsa-miR-373-3p	2.36	hsa-miR-211-5p	3.02	
hsa-miR-552-3p	1.89	hsa-miR-34b-5p	2.36	hsa-miR-375	3.76	
hsa-miR-382-5p	1.98	hsa-miR-9-3p	2.38	hsa-miR-205-5p	5.12	
hsa-miR-616-3p	2.00	hsa-miR-514a-3p	2.56			

No.: number of undetected samples; SD: standard deviation. ^†^These 51 genes were used to calculate the global mean. ^*^These 10 genes were selected as candidate reference genes.

## Results

### Candidate genes and stability analysis

**Figure 1:**
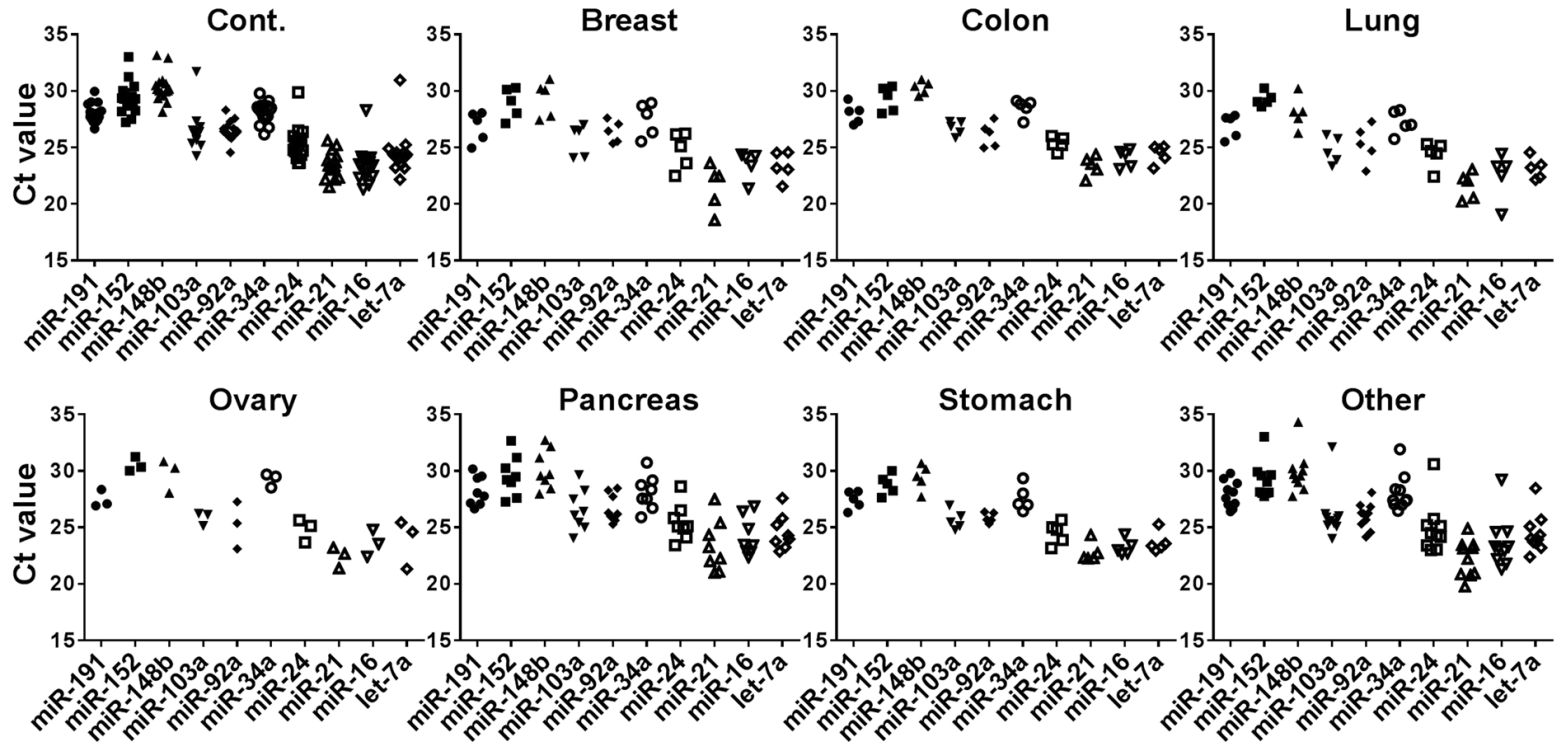
The threshold cycle (Ct) value of qRT-PCR for candidate reference genes in each sample type. These samples were formalin-fixed, paraffin-embedded lymph node tissues from non-cancerous (Cont.) and metastatic cancer tissues (Breast, Colon, Lung, Ovary, Pancreas, Stomach, and Other represent the location of primary tumor). The dot plots represents the raw Ct values of reference gene candidates.

Fifty-one out of 71 genes, which were expressed in more than 90% of the samples, were employed to calculate the internal reference value for the comparative ΔCt method with the global mean normalization strategy (ΔCt_Glo_) (Table 1). The top 10 endogenous reference gene candidates selected by the low variability of the ΔCt_Glo_ values were miR-16, miR-21, miR-24, miR-34a, miR-92a, miR-103a, miR-148b, miR-152, miR-191, and let-7a. The distribution of raw Ct values of the 10 candidate genes in control and cancer groups is shown in Fig. 1. After subdivision on the basis of the location of primary tumor, a specific trend was not observed on raw Ct values.

The expression levels of these candidate genes were evaluated using BestKeeper analysis in all samples (Table 2). Among all candidate genes, miR-103a exhibited the best correlation between the BestKeeper Index and the candidate gene (r = 0.963, p ≤ 0.001). This BestKeeper Index was calculated using only seven genes excluding miR-16, miR-21, and miR-148b owing to their unacceptable variability (SD > 1.05). BestKeeper analysis determined that miR-103a was the most stable gene by combining a low standard deviation and a high correlation.

**Table 2:** Descriptive statistics obtained by BestKeeper analysis.

Gene	miR-21	miR-16	miR-148b	miR-92a	miR-34a	miR-191	miR-152	let-7a	miR-24	miR-103a	BKI (n = 10)	BKI (n = 7)
N	57	57	57	57	57	57	57	57	57	57	57	57
GM [Ct]	22.733	23.492	29.789	26.157	27.999	27.741	29.281	24.124	25.017	26.058	26.138	26.573
AM [Ct]	22.786	23.543	29.825	26.182	28.026	27.762	29.311	24.170	25.059	26.102	26.165	26.598
Min [Ct]	18.592	19.075	26.276	22.905	25.543	24.954	27.126	21.330	22.417	23.362	23.983	24.757
Max [Ct]	27.486	29.219	34.338	28.479	31.928	30.182	33.021	30.964	30.615	32.121	30.087	30.484
SD [±Ct]	1.197	1.074	1.099	0.846	0.981	0.831	1.019	1.035	1.020	1.005	0.869	0.821
CV [%Ct]	5.255	4.562	3.685	3.231	3.500	2.994	3.478	4.280	4.070	3.852	3.320	3.086
r with BKI (n = 10)	0.830^*^	0.863^*^	0.914^*^	0.751^*^	0.785^*^	0.820^*^	0.842^*^	0.899^*^	0.929^*^	0.961^*^	—	—
r with BKI (n = 7)	—	—	—	0.745^*^	0.780^*^	0.801^*^	0.854^*^	0.923^*^	0.942^*^	0.963^*^	—	—
Ranking	10	9	8	7	6	5	4	3	2	1	—	—

AM: arithmetic; BKI: BestKeeper Index; Ct: threshold cycle value; CV: coefficient of variation; GM: geometric; Max: maximum value; Min: minimum value; N: number of available samples; n: number of genes; r: Pearson correlation coefficient; SD: standard deviation; *p ≤ 0.001, P-value associated with the Pearson correlation coefficient. BKI was calculated for 10 genes and for seven genes, excluding the gene with the highest standard deviation.

**Figure 2:**
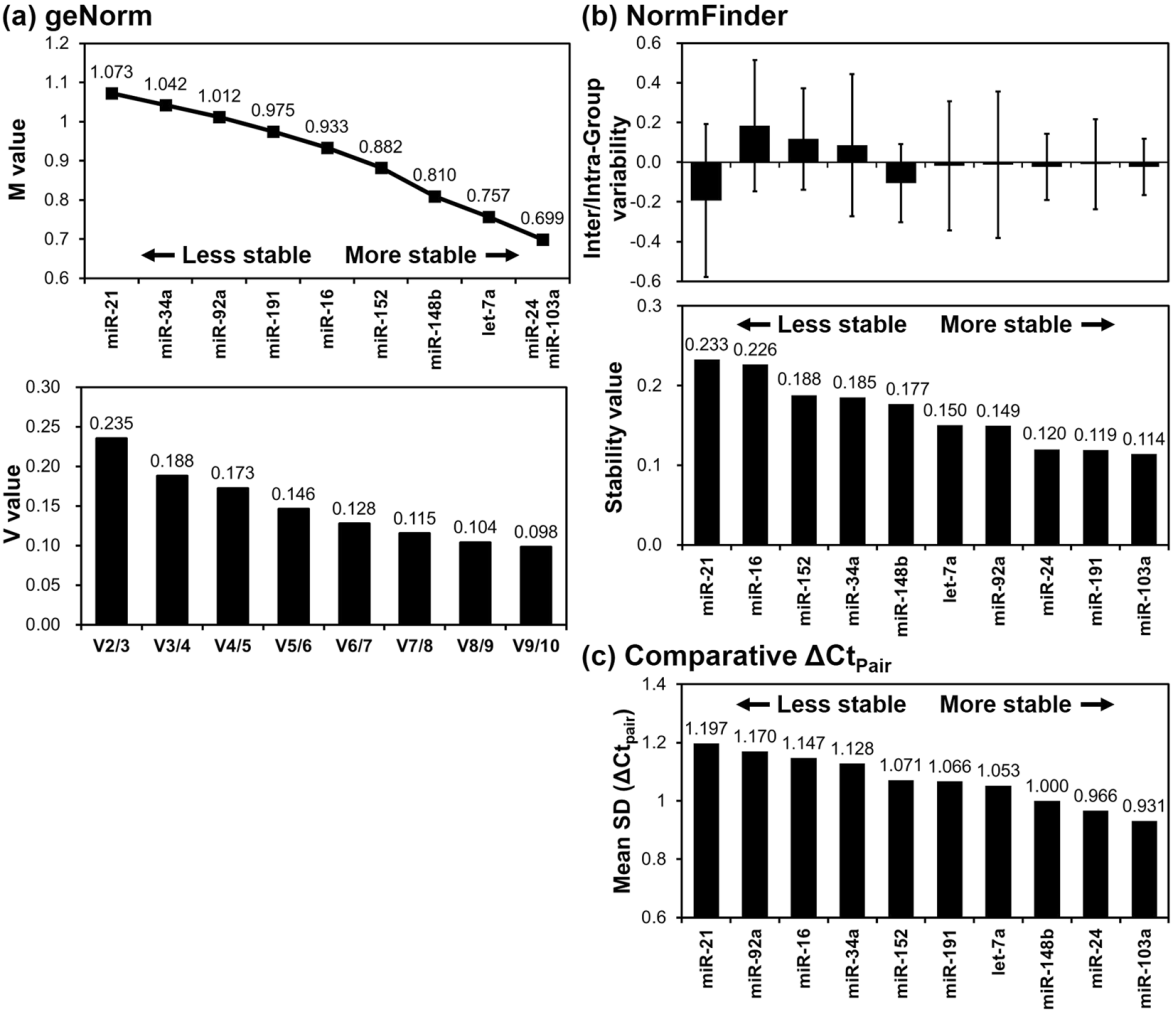
Stability analysis using geNorm, NormFinder, and the comparative ΔCt method. (**a**) The average gene expression stability (M value) and the pairwise variation value (V value) calculated by geNorm. (**b**) The inter- and intra-group variability and stability values estimated using NormFinder. In the variability graph, the error bars represent the average of the intra-group variability and the boxes indicate the confidence interval for the inter-group variability. (**c**) Mean standard deviation (Mean SD) was calculated by the comparative ΔCt method performing pairwise comparisons between genes (ΔCt_Pair_).

Using geNorm analysis, all 10 candidate genes had an M value below the recommended threshold of 1.5 (Fig. 2a). This analysis indicated that miR-24 and miR-103a were the most stable genes among the 10 candidate genes (M = 0.699). However, a highly stable gene should have an M value below reference value of 0.5. geNorm also identified the optimal number of reference genes in terms of the pairwise variation (V) between normalization factors. As shown in Fig. 2a, the use of the five most stable genes was recommend for optimal performance (V value < 0.15) in our study.

NormFinder was used to calculate stability values derived from the intra- and inter-group variability. This analysis revealed that among the 10 candidate genes, miR-103a was the most stable gene (Stability value = 0.114) in sample groupings from metastatic cancer and non-cancerous tissues (Fig. 2b). The best combination recommended by NormFinder was a set of miR-148b and miR-152 (Stability value = 0.087).

The comparative ΔCt method was used to identify the variability of all possible gene combinations within each sample. The stability of a gene was assessed by the mean of the standard deviations of the ΔCt values (ΔCt_Pair_) over the pairs including the gene. As shown in Fig. 2c, miR-103a was identified as the most stable gene (Mean SD = 0.931) in all the samples, followed by miR-24 (Mean SD = 0.966) and miR-148b (Mean SD = 1.000).

The ranking assessment of expression stability by the four analyses is reported in Table 3. Combining the results of the four analyses, the ranking assessment of expression stability using the geometric mean suggested that miR-103a was the most stable gene, followed by miR-24 and let-7a.

**Table 3:** Calculation of the overall stability according to the four stability analyses used in this study.

Rank	Gene	Overall Ranking	BestKeeper	geNorm	NormFinder	Comparative ΔCt_Pair_
			r with BKI (Ranking)	M value (Ranking)	Stability value (Ranking)	Mean SD (Ranking)
1	miR-103a	1.0	0.963 (1)	0.699 (1)	0.114 (1)	0.931 (1)
2	miR-24	1.9	0.942 (2)	0.699 (1)	0.120 (3)	0.966 (2)
3	let-7a	3.7	0.923 (3)	0.757 (3)	0.150 (5)	1.053 (4)
4	miR-191	4.3	0.801 (5)	0.975 (7)	0.119 (2)	1.066 (5)
5	miR-148b	4.9	−(8)	0.810 (4)	0.177 (6)	1.000 (3)
6	miR-152	5.6	0.854 (4)	0.882 (5)	0.188 (8)	1.071 (6)
7	miR-92a	6.7	0.745 (7)	1.012 (8)	0.149 (4)	1.170 (9)
8	miR-34a	7.2	0.780 (6)	1.042 (9)	0.185 (7)	1.128 (7)
9	miR-16	7.9	−(9)	0.933 (6)	0.226 (9)	1.147 (8)
10	miR-21	10.0	−(10)	1.073 (10)	0.233 (10)	1.197 (10)

BKI: BestKeeper Index, calculated for seven genes; SD: standard deviation.

### Stability analysis of the combined genes

NormFinder selected miR-148b and miR-152 as the best combination. In addition, based on the stability rankings shown in Table 3, we chose the combination of two (miR-24/miR-103a) or three (miR-24/miR-103a/let-7a) genes. The stability analysis of these gene sets was performed with the following normalization factors: global mean, miR-16, miR-191, miR-16/miR-345, miR-16/let-7a, and U6/SNORD44/SNORD48. Because U6, SNORD44, and SNORD48 exhibited very low stability compared with the five normalization factors, they were combined to avoid bias in the results. Briefly, U6, SNORD44, and SNORD48 were consistently ranked (8 th, 7 th, and 6 th, respectively) as the least stable genes by the four stability analyses, e.g., BestKeeper indicated a high standard deviation for U6 (1.837) and SNORD44 (1.315) and a low correlation for SNORD48 (r = 0.538).

In the BestKeeper analysis (Table 4), miR-24/miR-103a/let-7a exhibited the highest correlation coefficients with BestKeeper Index (r = 0.981). U6/SNORD44/SNORD48 and miR-16 were excluded from the correlation analysis due to its high standard deviation of >1.05 (Table 4).

**Table 4:** Descriptive statistics for combined genes obtained by BestKeeper analysis.

Gene	U6/SNORD44/SNORD48	miR-16	miR-191	miR-16/miR-345	miR-148b/miR-152	Global mean	miR-16/let-7a	miR-24/miR-103a	miR-24/miR-103a/let-7a	BKI (n = 9)	BKI (n = 7)
N	57	57	57	57	57	57	57	57	57	57	57
GM [Ct]	28.048	23.492	27.741	26.638	29.540	28.658	23.813	25.540	25.070	26.426	26.647
AM [Ct]	28.094	23.543	27.762	26.670	29.568	28.678	23.856	25.581	25.110	26.455	26.674
Min [Ct]	24.824	19.075	24.954	22.945	27.445	26.577	20.613	23.162	22.905	24.400	24.700
Max [Ct]	32.169	29.219	30.182	30.726	33.680	31.885	29.628	31.368	30.848	30.718	30.889
SD [±Ct]	1.277	1.074	0.831	0.931	0.954	0.822	1.015	0.987	0.971	0.889	0.872
CV [%Ct]	4.545	4.562	2.994	3.490	3.226	2.866	4.255	3.857	3.867	3.360	3.269
r with BKI (n = 9)	0.757^*^	0.910^*^	0.799^*^	0.899^*^	0.931^*^	0.951^*^	0.962^*^	0.968^*^	0.972^*^	—	—
r with BKI (n = 7)	—	—	0.808^*^	0.897^*^	0.947^*^	0.955^*^	0.958^*^	0.975^*^	0.981^*^	—	—
Ranking	9	8	7	6	5	4	3	2	1	—	—

AM: arithmetic; BKI: BestKeeper Index; Ct: threshold cycle value; CV: coefficient of variation; GM: geometric; Max: maximum value; Min: minimum value; N: number of available samples; n: number of factors; r: Pearson correlation coefficient; SD: standard deviation; *p ≤ 0.001, P-value associated with the Pearson correlation coefficient. BKI was calculated for 9 factors and for seven factors, excluding the gene with the highest standard deviation.

**Figure 3:**
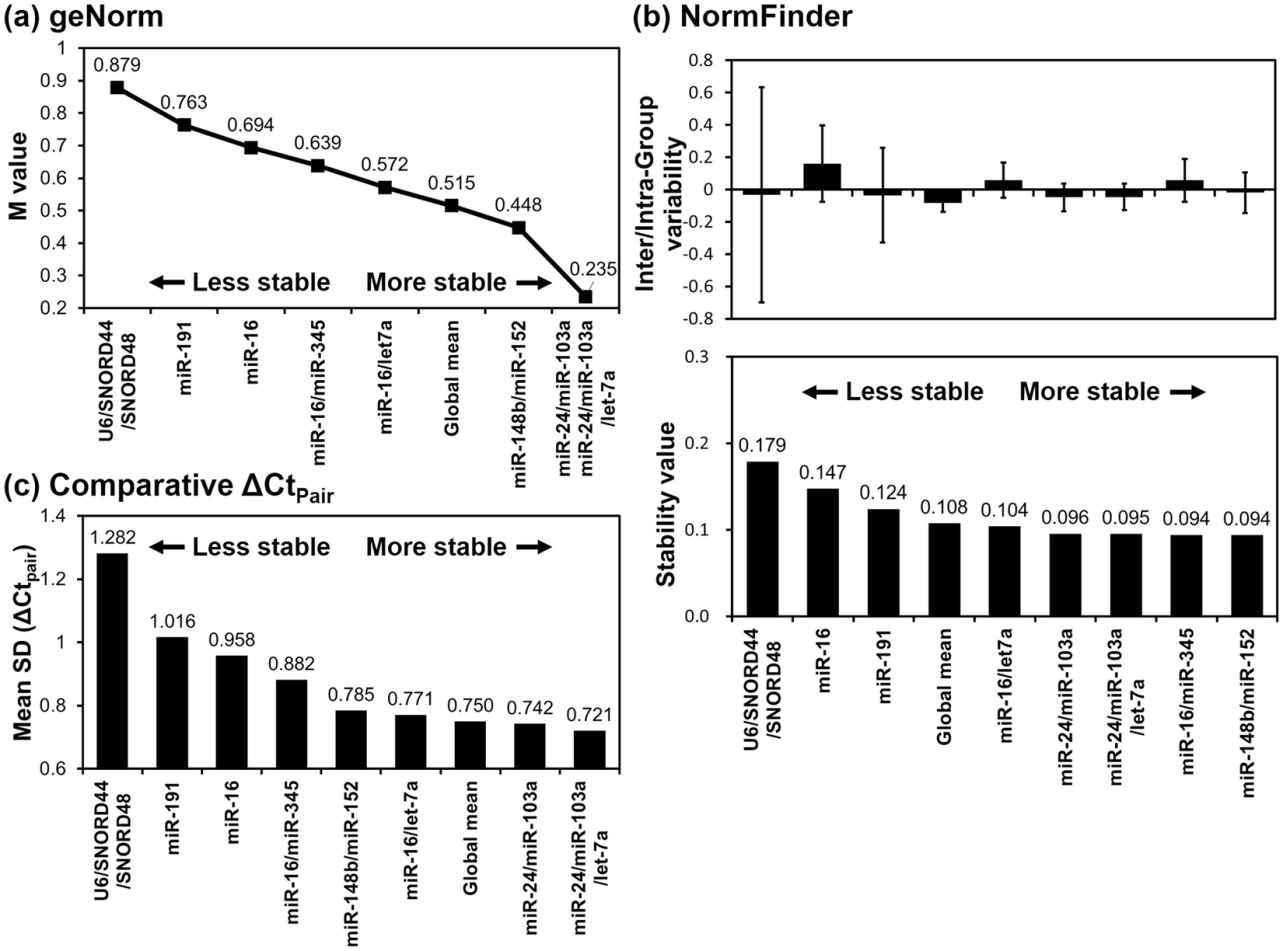
Expression stability of endogenous reference gene candidates determined by geNorm, NormFinder, and the comparative ΔCt_Pair_ method. (**a**) The M values and V values calculated by geNorm. (**b**) The inter- (box) and intra- (error bars) group variability and stability values determined using NormFinder. (**c**) Mean standard deviation (Mean SD) was calculated by the comparative ΔCt_Pair_ method.

The M values of miR-24/miR-103a and miR-24/miR-103a/let-7a calculated by geNorm were lower than 0.5 (Fig. 3a); therefore, these combinations were suggested as suitable endogenous references. NormFinder revealed that miR-148b/miR-152 had minimal inter- and intra-group variability, with a stability value of 0.094 (Fig. 3b). The results of the comparative ΔCt_Pair_ method demonstrated that miR-24/miR-103a/let-7a and miR-24/miR-103a had the lowest variability with standard deviations of 0.721 and 0.742, respectively (Fig. 3c).

The overall results of the stability analysis obtained using the statistical tools showed that miR-24/miR-103a/let-7a ranked as the most stable gene combination, whereas U6/SNORD44/SNORD48 was the least stable combination (Table 5).

**Table 5:** Stability ranking of the endogenous reference gene candidates.

Rank	Gene	Overall Ranking	BestKeeper	geNorm	NormFinder	Comparative ΔCt_Pair_
			r with BKI (Ranking)	M value (Ranking)	Stability value (Ranking)	Mean SD (Ranking)
1	miR-24/miR-103a/let-7a	1.3	0.981 (1)	0.235 (1)	0.095 (3)	0.721 (1)
2	miR-24/miR-103a	2.0	0.975 (2)	0.235 (1)	0.096 (4)	0.742 (2)
3	miR-148b/miR-152	2.9	0.947 (5)	0.448 (3)	0.094 (1)	0.785 (5)
4	Global mean	4.1	0.955 (4)	0.515 (4)	0.108 (6)	0.750 (3)
5	miR-16/let-7a	4.2	0.958 (3)	0.572 (5)	0.104 (5)	0.771 (4)
6	miR-16/miR-345	4.6	0.897 (6)	0.639 (6)	0.094 (2)	0.882 (6)
7	miR-16	7.5	−(8)	0.694 (7)	0.147 (8)	0.958 (7)
8	miR-191	7.5	0.808 (7)	0.763 (8)	0.124 (7)	1.016 (8)
9	U6/SNORD44/SNORD48	9.0	−(9)	0.879 (9)	0.179 (9)	1.282 (9)

BKI: BestKeeper Index; SD: standard deviation.

### Effects of normalization on relative quantification

We investigated the impact of normalization on the expression of miR-29c in a metastatic lymph node with colon cancer in comparison with that in a lymph node without cancer.

**Figure 4:**
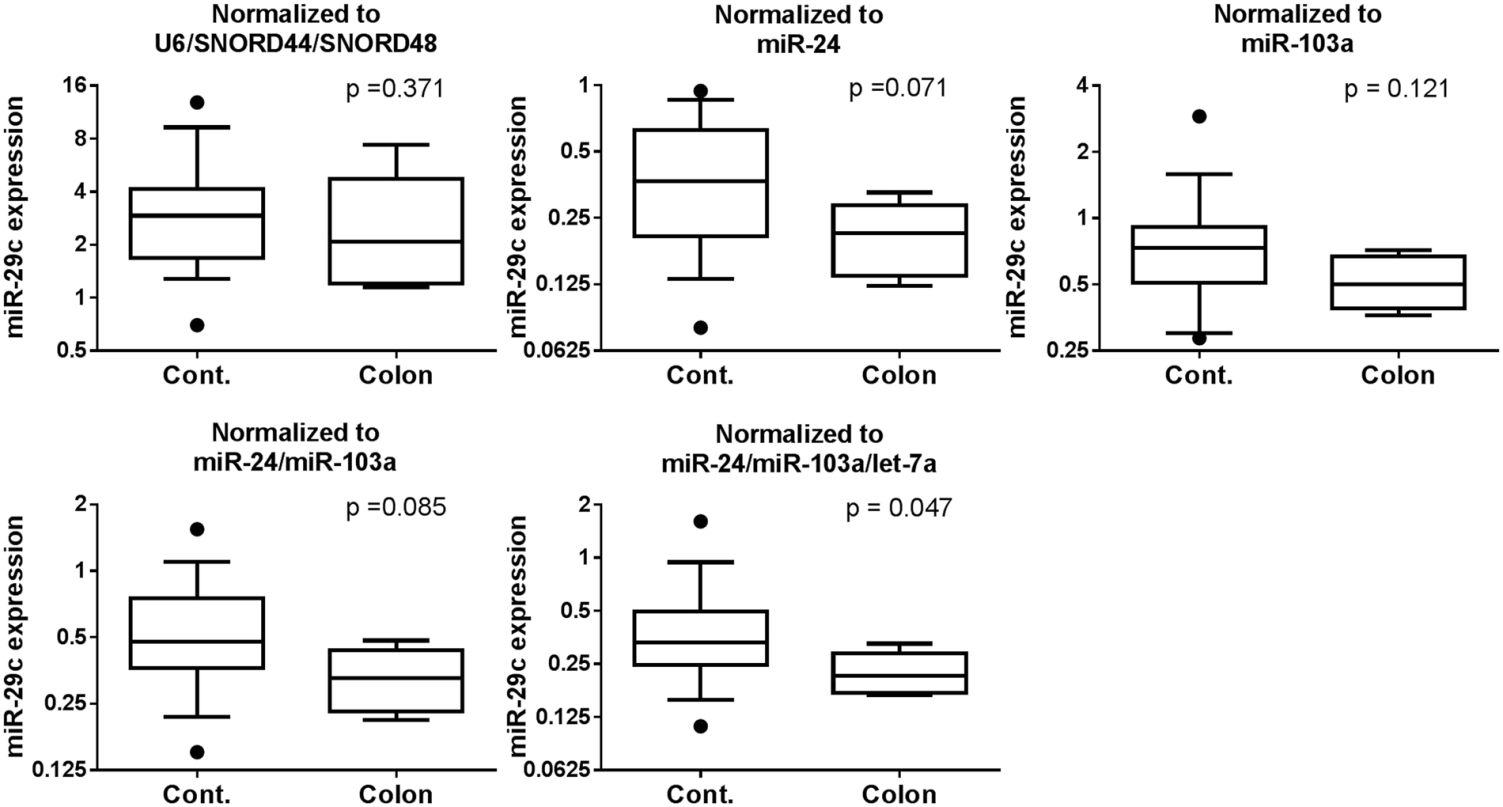
Effect of normalization on the expression of miR-29c in formalin-fixed, paraffin-embedded lymph node tissue. Relative expression of a metastatic lymph node (Colon: n = 5) was compared with a lymph node without cancer (Cont.: n = 16). The expression levels (2^−ΔCt^) of miR-29c are shown as box plots (log2 scale on the y-axis). The median is represented as a horizontal line within each box and its lower and upper edges represent the 25–75 percentile region. The whiskers represent the 10–90 percentile region, and the dots represent the outliers. Statistically significant differences were determined using the Mann–Whitney *U* test (two-tailed test).

Figure 4 shows the expression of miR-29c normalized to each of the five normalization factors: miR-24, miR-103a, miR-24/miR-103a, miR-24/miR-103a/let-7a and U6/SNORD44/SNORD48.

The expression of miR-29c normalized to miR-24/miR-103a/let-7a significantly decreased in a metastatic lymph node with colon cancer in comparison with that in a lymph node without cancer (p < 0.05). However, this difference was not detected when the expression of miR-29c was normalized to the other four factors.

## Discussion

In this study, we presented the use of the mean Ct value of miR-24, miR-103a, and let-7a as a suitable normalization factor for miRNA expression in FFPE lymph node tissue using qRT-PCR.

qRT-PCR is the gold standard method for miRNA quantitation because it is the most sensitive and reproducible method. However, the accuracy of results depends on the selected normalization factor. To obtain accurate data using qRT-PCR, many studies have identified stable reference genes in tissues other than lymph node, such as SNORD48 for atrial or organ tissue samples^17,18^, miR-16/let-7a for breast tissue samples^10^, and miR-16/miR-345 for colorectal tissue samples^19^. Moreover, the use of a single reference gene, unless fully validated, is insufficient to obtain reliable miRNA expression^20,21^.

In fact, we had planned to identify the origin of the primary tumor using miRNA expression patterns in metastatic lymph node cells using qRT-PCR at first. However, we noticed that an endogenous reference gene for miRNA expression analysis had not been identified in FFPE lymph node with metastatic cancer. Prior to miRNA expression analysis, a suitable endogenous reference gene should be evaluated to avoid misinterpretation of data and identify true changes in miRNA expression levels^7^. Therefore, we validated that the combination of three miRNAs could be used as a suitable endogenous reference in a lymph node with metastatic cancer for qRT-PCR. This miRNA combination is not surprising because miR-24, miR-103a, and let-7a have been reported to exhibit a high expression stability in tumor tissue^3,10–12^. This suitable set of endogenous reference genes was determined based on the combination of four statistical approaches: BestKeeper, geNorm, NormFinder, and the comparative ΔCt method. These statistical analyses have been developed to identify optimal endogenous reference genes in a given set of samples. For the selection of candidate reference genes, we used the global mean normalization across 51 genes (the comparative ΔCt_Glo_ method). The global mean normalization can be used when a large number of miRNAs (typically more than 50) are analyzed in a sample. In addition, this normalization assumes that the mean expression of all miRNAs in a sample is constant. The Ct values of all 51 genes in a sample had normal distribution (Shapiro–Wilk test; p > 0.1 for all samples), which allowed the use of the global mean normalization method in our study. As mentioned in Methods, BestKeeper considers standard deviation and correlation of Ct values to assess stability^13^, whereas geNorm uses a pairwise comparison approach and assumes that the expression ratio of two ideal endogenous reference genes is identical in all samples, regardless of experimental conditions^14^. geNorm also calculates the optimal number of reference genes. It recommended the use of five reference genes for optimal normalization performance. However, we restricted our analysis to the combination of up to three genes, because we considered that the use of a larger number of reference genes may be impractical in further studies, such as the identification of tissue origin in cancers of unknown primary. NormFinder can account for heterogeneity among sample groups as it estimates intra- and inter-group variability^15^. In addition, NormFinder also allows the calculation of the stability values for a set of genes. It is important to note an imbalance of the number of samples between the control and the cancer groups. This imbalance may have an effect on NormFinder analysis, where balanced populations are generally requested. The comparative ΔCt_Pair_ method compares relative expression of ‘pairs of genes’ within each sample for all candidate gene combinations^16^.

The stability of selected gene sets were evaluated using the other normalization factors (i.e., global mean, miR-16, miR-191, miR-16/miR-345, miR-16/let-7a, U6/SNORD44/SNORD48). These factors are commonly used as reference for miRNA quantitation in cancer tissue^7,10,12,19^. Based on the initial analysis, we combined U6, SNORD44, and SNORD48. These three genes are well-known reference genes in miRNA analysis, however, their use has been reported to involve high variability in some experimental conditions^12^.

Consequently, these multiple analyses revealed that the combination of miR-24/miR-103a/let-7a was the most stable endogenous normalization factor in our experimental conditions. miRNA expression stability must be carefully assessed in each specific experimental setting, because it is also possible that these miRNAs might be also associated with lymph node metastasis. In fact, the aberrant expression of miR-24 (higher expression) and let-7a (lower expression) in breast cancer is shown to play a key role in tumor invasion and metastasis^22,23^. Therefore, a combination of three miRNAs has an important meaning to avoid erroneous results.

We also quantified the expression levels of miR-29c in a metastatic lymph node with colon cancer to evaluate the normalization efficiency by four different endogenous factors. A significant downregulation of miR-29c expression was observed in the metastatic lymph node when data were normalized to the combination of miR-24/miR-103a/let-7a. The downregulation of miR-29c expression has been reported in various cancers including colon cancer^24,25^. Although the function of miR-29c expression in colon cancer has not been clarified, miR-29c exhibits a tumor suppressor function in several types of cancer by targeting TNFAIP3 and cyclin E, thereby inhibiting tumorigenesis and metastasis^25–27^. Thus, the use of the other normalization factors, which did not reveal the significant downregulation of miR-29c, should be discouraged for FFPE lymph node studies because it may lead to incorrect results.

To our knowledge, this is the first study to determine reference genes in FFPE lymph node tissues from patients with different kinds of cancer. Our result suggests the importance of assessing the gene stability for each experimental condition.

We acknowledge some limitations of the present study as follows: the relatively small sample size; the qRT-PCR assay based on SYBR Green, which is not so specific as the TaqMan method; the absence of replicates in the determination of Ct values; and the fact that the analysis of combined genes involved repeated elements (e.g., miR-24/miR-103a and miR-24/miR-103a/let-7a) with potential correlations among sets, which may have affected the analyses. Although this study assessed the stability of 71 genes including the most commonly used reference genes, more suitable reference gene combinations may be identified in the future. Our results should be applied to FFPE lymph node tissue in humans. Therefore, it does not eliminate the use of common reference genes in other experimental conditions.

## Conclusion

We identified a suitable endogenous reference that can be used to study miRNA expression in FFPE lymph node tissue from patients with metastatic cancer. This result will provide valuable information for future miRNA expression studies in FFPE lymph node tissue samples with metastatic cancer.

## Methods

### Tissue samples and ethical statement

FFPE lymph node tissue samples were obtained from 41 metastatic cancer and 16 non-cancerous tissues at Ibaraki Prefectural Central Hospital, Japan. The characteristics of the samples are presented in Table 6.

**Table 6:** Characteristics of the samples.

Sample Group	Location of primary tumor	N (F; M)	Mean age (Range)	Subtype (N)
Metastatic LN	Breast	5	58	Invasive carcinoma (5)
		(5; 0)	(41–81)	
	Colon	5	68.6	Adenocarcinoma (5)
		(1; 4)	(56–75)	
	Lung	5	66.4	Small cell carcinoma (1), Squamous cell carcinoma (1), Adenocarcinoma (3)
		(4; 1)	(57–76)	
	Ovary	3	69.3	Squamous cell carcinoma (1), Adenocarcinoma (2)
		(3; 0)	(65–76)	
	Pancreas	8	67.1	Carcinoma (1), Adenocarcinoma (7)
		(2; 6)	(57–77)	
	Stomach	5	69.8	Adenocarcinoma (5)
		(2; 3)	(62–79)	
	Other	10	75.4	Carcinoma (not specified) (3), Small cell carcinoma (1), Squamous cell carcinoma (1), Adenocarcinoma (3), Melanoma (2)
		(5; 5)	(58–90)	
	Total	41	69	Carcinoma (9), Small cell carcinoma (2), Squamous cell carcinoma (3), Adenocarcinoma (25), Melanoma (2)
		(22; 19)	(36–90)	
Non-cancerous LN	—	16	76	Reactive LN without any tumor cells (16)
		(8; 8)	(51–83)	

F: female; LN: lymph node; M: male; N: number of samples; “Other” includes endometrial, thyroid, esophagus, and prostate cancers; cholangiocellular carcinoma; and malignant melanoma. The non-cancerous samples were mainly obtained from lymph node dissection and without any findings of cancer metastasis at histological analysis. They are from patients with lung cancer (5), bile duct cancer (3), endometrial cancer (2), colon cancer (1), hypopharynx cancer (1), malignant melanoma (1), parotid cancer (1), stomach cancer (1), and reactive mesenteric lymph node of ulcer of the small intestine (1).

This study was approved by the institutional review board of Ibaraki Prefectural Central Hospital, Japan. Informed consent was obtained for the use of archived clinical specimens, and all experimental methods were performed in accordance with the relevant guidelines and regulations.

### Total RNA isolation from FFPE lymph node tissue

Total RNA was extracted from four FFPE sections of 10-µm thickness using miRCURY^™^ RNA Isolation Kit (Exiqon, Vedbæk, Denmark) in accordance with the manufacturer’s instructions. RNA concentrations were measured using a NanoDrop ND-2000 spectrophotometer (NanoDrop Technologies, Wilmington, DE, USA). Only RNA samples with a 260/280 ratio of ≥1.8 were used.

### Quantitative real-time PCR

Total RNA (10 ng) isolated from FFPE tissue samples were reverse transcribed using miRCURY LNA Universal RT cDNA synthesis kit II with RNA spike-in (Exiqon) in a 10 µL reaction volume for 60 min at 42 °C and 5 min at 95 °C, according to the manufacturer’s instructions. The reverse transcription product (cDNA) was diluted 100-fold and quantified by qRT-PCR using an ExiLENT SYBR Green master mix (Exiqon). qRT-PCR was performed in custom-made 96-well Pick & Mix microRNA PCR panel plates (Exiqon) and a 7500 Fast Dx Real-Time PCR System (Applied Biosystems, Foster City, CA, USA). This PCR panel plates consisted of the 71 candidate genes (a single well for each gene) and the following quality controls: UniSp3 (triplicate), UniSp6 (single), and cel-miR-39 (single). The PCR protocol was applied as follows: incubation for 10 min at 95 °C, followed by 40 cycles of 10 s at 95 °C and 1 min at 60 °C, with a final melting curve analysis. The Ct values for qRT-PCR were determined using the SDS v1.4 software (Applied Biosystems) and the single-threshold method. Ct values that displayed unusual amplification curves (e.g., low amplification efficiency) were excluded from further analysis.

### Selection of candidate genes and stability analysis

Ten candidate genes were selected based on the comparative ΔCt method with the global mean as the endogenous control. The global mean value was calculated as the mean Ct value of the genes detected in a sample. Only highly expressed genes (expressed in more than 90% of the samples) were selected for the global mean method. For these genes, the normal distribution of Ct values was tested by Shapiro–Wilk test. Global mean normalization has been demonstrated to be one of the highly accurate approaches for miRNA expression analysis^20^.

The ΔCt_Glo_ value was calculated by the following equation: ΔCt_Glo_ = Target gene Ct − Global mean Ct. In this analysis, gene stability was assessed by the standard deviation of the ΔCt_Glo_ values. The standard deviation was given for the variability in ΔCt_Glo_ values in all samples.

BestKeeper^13^ analysis determines the most stably expressed gene based on the Pearson correlation coefficient (r) of the BestKeeper Index, which is the geometric mean of Ct values of candidate genes. Based on the raw Ct values of each sample, standard deviation was calculated, and high standard deviations (>1) were considered inadequate.

geNorm^14^ analysis evaluates the stability of candidate reference genes based on the average pairwise variation of a gene compared with that of all other genes. Next, it identifies the optimal number of reference genes required by analyzing the pairwise variation (Vn/n + 1) among candidate genes. The gene stability value (M value) and the pairwise variation value (V value) were calculated with the geNorm software using 2^−ΔCtMin^ values. The ΔCt_Min_ value was calculated by the following equation: ΔCt_Min_ = Target gene Ct − Min Ct, where Min Ct is the lowest Ct value of a candidate gene. The lowest M value indicates the most stable expression, and values under 0.5 indicate an acceptably stable expression. The number of reference genes was considered as optimal when the V value was below 0.15.

NormFinder^15^ analysis is based on an ANOVA model that considers intra- and inter-group variability to evaluate the expression stability of a candidate gene. In this study, expression variations between the metastatic cancer and non-cancerous tissue were focused on. Exponentially transformed data (2^−Ct^ value) were used as input data in the NormFinder software. The lowest stability value indicates the most stably expressed gene.

In the comparative ΔCt method^16^, we calculated the ΔCt_Pair_ values for each pair of reference gene candidates, and assessed the stability of each gene using the mean of the standard deviations obtained from all the pairwise comparisons including the gene.

The overall stability ranking of candidate genes was determined using the geometric mean of the rankings generated from all four analyses.

### Assessment of stability for combined genes

In addition to the best reference gene, NormFinder identified also the best combination of two genes. In addition, based on the overall stability ranking, combinations of two or three genes were determined as endogenous reference candidates. To evaluate the stability of combined genes, stability analyses were performed in combination with other normalization factors (i.e., global mean, miR-16, miR-191, miR-16/miR-345, miR-16/let-7a, U6 snRNA, SNORD44, and SNORD48).

### Effect of normalization

To evaluate the effectiveness of the endogenous reference chosen in this study, the expression levels of miR-29c was measured using various reference genes. Relative expression levels were reported as 2^−ΔCt^. The Mann–Whitney *U* test was used to determine statistically significant differences in expression levels between the metastatic cancer and non-cancerous tissue. Statistical analysis was performed with GraphPad Prism 6.03 (GraphPad Software, San Diego, CA, USA). P-values of <0.05 were considered statistically significant.

The datasets generated during and/or analyzed during the current study are available from the corresponding author on reasonable request.
